# Pyrido[3,4-*d*]pyrimidin-4(3*H*)-one metabolism mediated by aldehyde oxidase is blocked by C2-substitution

**DOI:** 10.1080/00498254.2016.1230245

**Published:** 2016-10-26

**Authors:** Angela Hayes, N. Yi Mok, Manjuan Liu, Ching Thai, Alan T. Henley, Butrus Atrash, Rachel M. Lanigan, Jimmy Sejberg, Yann-Vaï Le Bihan, Vassilios Bavetsias, Julian Blagg, Florence I. Raynaud

**Affiliations:** Cancer Research UK Cancer Therapeutics Unit, The Institute of Cancer Research, London, UK

**Keywords:** Aldehyde oxidase, metabolism, pyrido[3,4-d]pyrimidin-4(3H)-one

## Abstract

1. We have previously described C8-substituted pyrido[3,4-*d*]pyrimidin-4(3*H*)-one derivatives as cell permeable inhibitors of the KDM4 and KDM5 subfamilies of JmjC histone lysine demethylases.

2. Although exemplar compound **1** exhibited moderate clearance in mouse liver microsomes, it was highly cleared *in vivo* due to metabolism by aldehyde oxidase (AO). Similar human and mouse AO-mediated metabolism was observed with the pyrido[3,4-*d*]pyrimidin-4(3*H*)-one scaffold and other C8-substituted derivatives.

3. We identified the C2-position as the oxidation site by LC-MS and ^1^H-NMR and showed that C2-substituted derivatives are no longer AO substrates.

4. In addition to the experimental data, these observations are supported by molecular modelling studies in the human AO protein crystal structure.

## Introduction

Methylation and demethylation of lysine residues on the histone H3 tail constitute important epigenetic modifications that differentiate transcriptionally active and inactive chromatin (Kooistra & Helin, 2012; Shiau et al., 2013). These processes are highly deregulated in cancer offering opportunities for drug discovery and development (Helin & Dhanak, 2013; Hoffmann et al., 2012; Lohse et al., 2011). It has been demonstrated that the KDM4 and KDM5 subfamilies of Jumonji C (JmjC) histone lysine demethylases are involved in oncogenesis and drug resistance and their overexpression is commonly observed in various human tumour types (Berry & Janknecht, 2013; Blair et al., 2011; Kawazu et al., 2011; Li et al., 2011; Liu et al., 2009; Roesch et al., 2013; Sharma et al., 2010; Walters et al., 2014, Yang et al., 2015; Young & Hendzel, 2013). Cell penetrant inhibitors of the KDM4 and KDM5 subfamilies have been reported (Westaway et al., 2016), and we are currently evaluating the potential of dual KDM4 and KDM5 subfamily small-molecule pyrido[3,4-*d*]pyrimidin-4(3*H*)-one-based inhibitors as anticancer agents (Bavetsias et al., 2016).

In addition to evaluating the potency of our inhibitors on various JmjC histone lysine demethylases, our assay cascade includes the assessment of their metabolic stability in liver microsomes. Our current assay evaluates clearance by CYP450 and UGT enzymes, which metabolise the majority of xenobiotics. Once compound stability in mouse liver microsomes has been established, we evaluate the *in vivo* pharmacokinetics in mice following iv and po administration to select compounds suitable for further pharmacokinetic–pharmacodynamic testing.

We have previously described C8-substituted pyrido[3,4-*d*]pyrimidin-4(3*H*)-one-based JmjC histone demethylase inhibitors as potent and cell permeable KDM4/KDM5 subfamily inhibitors (Bavetsias et al., 2016). Exemplar compound **1** exhibited low clearance in mouse liver microsomes, but very high *in vivo* clearance.

In the current study, we characterised the metabolic route of **1** in mouse and human cytosolic fractions and established the nature and the position of metabolism. These experimental observations led to the hypothesis that the pyrido[3,4-*d*]pyrimidin-4-one scaffold is liable to C2-oxidation by aldehyde oxidase (AO). The susceptibility of the scaffold to AO metabolism was confirmed experimentally. In addition, we showed that C2-substituted pyrido[3,4-*d*]pyrimidin-4-one derivatives are protected from oxidation by AO. Further molecular modelling studies in the human AO protein crystal structure suggested a ligand-binding mode that is consistent with our experimental observations regarding metabolic liability.

## Material and methods

### Compound synthesis

The syntheses of **1** and close analogue **2** have been previously reported (Bavetsias et al., 2016). Compound **3** is commercially available. Compounds **4**, **5** and **6** were prepared by methodologies analogous to those reported for the synthesis of C2-unsubstituted pyrido[3,4-*d]*pyrimidin-4-one derivatives (Bavetsias et al., 2016). Detailed experimental data including compound characterisation is provided in the Supporting Information.

### Microsomal and cytosolic clearance studies

Liver microsomes and cytosolic fractions derived from female CD1 mice (MLM), female Sprague-Dawley rats (RLM), a pool of 50 mixed gender human donors (HLM) and mouse and human liver cytosol were obtained from Tebu Bio (Le Perrey-en-Yvelines, France). CYP450 and AO-mediated metabolism of compounds (1 μM DMSO) were assessed by incubation with liver microsomes and cytosol (0.5 mg/ml) respectively in 0.1 M PBS at 37 °C. Microsomal incubations were initiated by addition of NADPH (1 mM), UDPGA (2.5 mM) and MgCl_2_ (3 mM) for phase I and II reactions. Control incubations were generated by the omission of NADPH and UDPGA from the incubation reaction. Cytosol incubations were carried out at pH 7.4 in the presence of 0.1 mM EDTA. They were initiated by addition of 1 μM compound in the presence and absence of selective AO inhibitor raloxifene and stopped by addition of 3 volumes of acetonitrile at 0, 5, 10, 15 and 30 min, then centrifugation at 3000 rpm for 30 min at 4 °C. Supernatant was analysed by LC-MS/MS to determine clearance. We also tested the effect of the selective xanthine oxidase inhibitor allopurinol on the cytosolic clearance of compound **1**.

### *In vivo* mouse pharmacokinetic studies

*In vivo* experiments were conducted according to the UK guidelines for the welfare and use of animals in cancer research UKCC (Workman et al., 2010). Female Balb/c mice (*n* = 3 per route) received either a single intravenous (bolus) injection or a single oral administration (by gavage) of compound **1** (Figure 4) (5 mg/kg) in 10% DMSO, 0.1% Tween20 in Saline. Samples of whole blood at specific time points were spotted onto Whatman FTA cards (GE Healthcare, United Kingdom), allowed to dry at ambient temperature for at least 2 hours. Compound **1** was quantified by 6 mm punch outs and extracted using acetonitrile as previously described (Roberts et al., 2016).

### Mass spectrometric analysis

*In vitro* microsomal incubation and *in vivo* samples were analysed using a Waters Xevo TQ-S mass spectrometer by multiple reaction monitoring (Waters, Milford, MA). Conditions were 0.2% formic acid (mobile phase A) and acetonitrile (mobile phase B). Separation was achieved on a Phenomenex Kinetex C18 column (2.1 × 50 mm; 2.6 μm). The column was equilibrated at initial condition of 95% A and 5% B for 0.5 min, linear gradient over 3 min to 100% B, held over 1 min, followed by linear gradient back to 5% B over 0.1 min, at 0.6 mL/min flow rate. AO incubation samples were analysed for metabolites of **1** using an Agilent 6520 QTOF MS (Agilent, Santa Clara, CA). Using the above mobile phases, separation was achieved on a Phenomenex Kinetex C18 column (2.1 × 100 mm; 2.6 μm) (Phenomenex, Torrance, CA). The column was equilibrated at initial condition of 95% A and 5% B (0.5 min), linear gradient (15 min) to 50% B, held for 1 min, followed by linear gradient back to 5% B (0.5 min), at 0.6 mL/min flow rate.

### Liquid chromatographic separation

Standard injections (with needle wash) of the sample were made onto a ZORBAX Eclipse XDB-C18 column (4.6 × 150 mm, 5 μm). Chromatographic separation was carried out at 30 °C using a 1260 Series HPLC (Agilent, Santa Clara, CA) over gradient elution at a flow rate of 1.5 mL/min. UV-Vis spectra were acquired at 254 nm on a 1260 Series diode array detector (Agilent, Santa Clara, CA). Fractions were collected using an Agilent analytical scale fraction collector.

### Structural identification of the metabolites by ^1^H NMR

^1^H NMR data was collected on a Bruker Avance 500 spectrometer equipped with a 1.7 mm TXI probe (Bruker, Billerica, MA). The ^1^H NMR spectrum was referenced to the internal deuterated solvent. The operating frequency for ^1^H was 500 MHz. All NMR data were acquired at the temperature of 295 K. All data were acquired and processed using Bruker Topspin 2.1.

### Aldehyde oxidase ligand-binding predictions

The protein–ligand co-crystal structure of human aldehyde oxidase (hAOX1) (PDB code: 4uhw) (Coelho et al., 2015) was prepared using Protein Preparation Wizard (Maestro v9.3, Schrödinger, LLC: New York, NY). To predict proposed binding modes of the ligands, Glide (Grid-based Ligand Docking with Energetics) (Glide v5.8, Schrödinger, LLC: New York, NY) was used for the docking experiments. The receptor grid was defined by a grid box of 30 × 30 × 30 Å^3^ with a default inner box (10 × 10 × 10 Å^3^) centred on the molybdenum cofactor (MoCo) in the hAOX1 catalytic domain in structure PDB 4uhw.

Ligands were prepared using LigPrep, applying the OPLS_2005 force-field with possible tautomeric states of neutral species within pH range 5.0–9.0 generated using Epik metal binding state settings (Ligprep v2.5, Schrödinger, LLC: New York, NY). Using Extra Precision (XP) settings, flexible docking of compound **3** was performed unconstrained. The predicted binding pose of compound **3** within the hAOX1 catalytic domain was subsequently used as the core coordinates constraint to evaluate the feasibility of other synthesised ligands adopting the same predicted binding mode.

## Results

**Figure 1.**
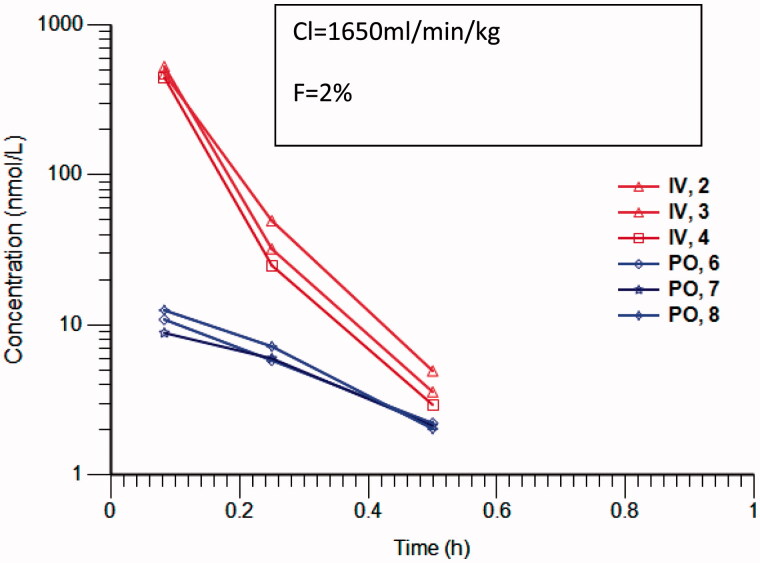
Concentration versus time profile of Compound **1** following 5mg/kg iv (red) and po (blue) in 10% DMSO, 1% tween 80 in saline. Profiles are from individual mice (*n* = 3). Analysis was carried out by LC-MS/MS with selected reaction monitoring. Clearance and bioavailability have been calculated with Phoenix non-compartmental analysis.

Exemplar pyrido[3,4-*d]*pyrimidin-4-one derivative **1** displayed moderate clearance in mouse and human liver microsomes (45.4 and 34.1 μL/min/mg, respectively) and low clearance in rat liver microsomes (10.4 μL/min/mg), which prompted us to investigate the pharmacokinetics of this compound in mice. Following iv and po administration of 5 mg/kg of compound **1** to female Balb C mice, the blood clearance was greater than hepatic blood flow with only 2% oral bioavailability (Figure 1). The measured mouse cytosolic clearance of **1** was 462.5 μL/min/mg, consistent with the observed high *in vivo* blood clearance. Addition of raloxifene, an inhibitor of aldehyde oxidase, to the cytosolic incubation greatly reduced the cytosolic clearance of compound **1** to 19 μL/min/mg, indicating that compound **1** is a substrate of mouse aldehyde oxidases. Compound **1** was also highly metabolised in human cytosol with a clearance of 752 μL/min/mg, which was reduced to 5 μL/min/mg by the addition of raloxifene. Addition of allopurinol to the cytosolic incubation did not affect the clearance showing that xanthine oxidase does not contribute to the cytosolic clearance of compound 1(data not shown).

**Figure 2.**
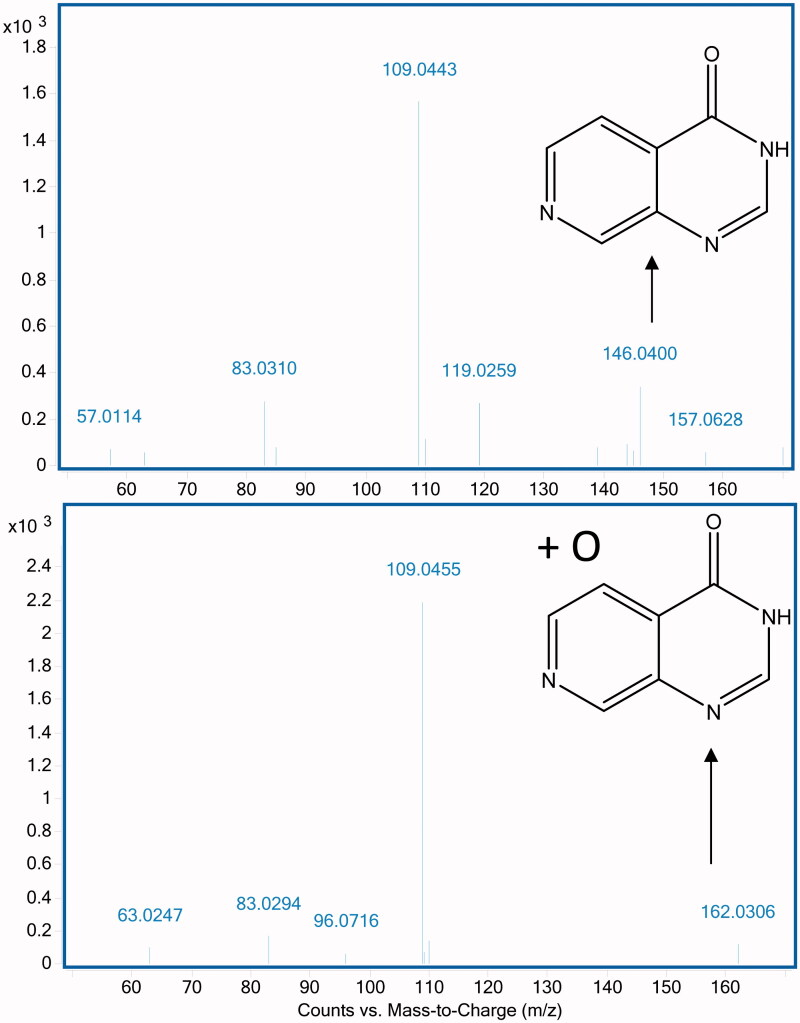
Fragmentation pattern of Compound **1** (m/z 379.17) and its oxidation product (m/z 395.17). The core pyrido[3,4-*d*]pyrimidin-4(3*H*)-one fragment in **1** (m/z 146.04) is oxidised in the metabolite (m/z 162.03). No further fragmentation was observed.

**Figure 3.**
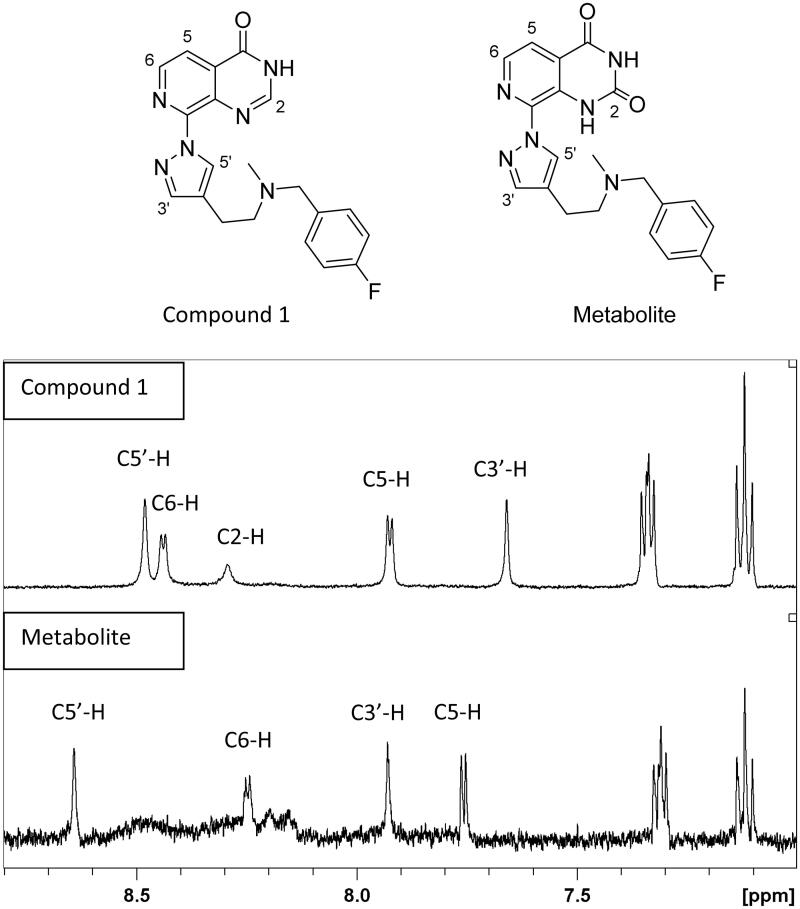
^1^H NMR spectrum of compound **1** and its oxidation product referenced to internal deuterated solvent. Protons C5 and C6 are still present in the metabolite suggesting that the oxidation is at position C2 of the pyrido[3,4-*d*]pyrimidin-4(3*H*)-one scaffold.

**Figure 4.**
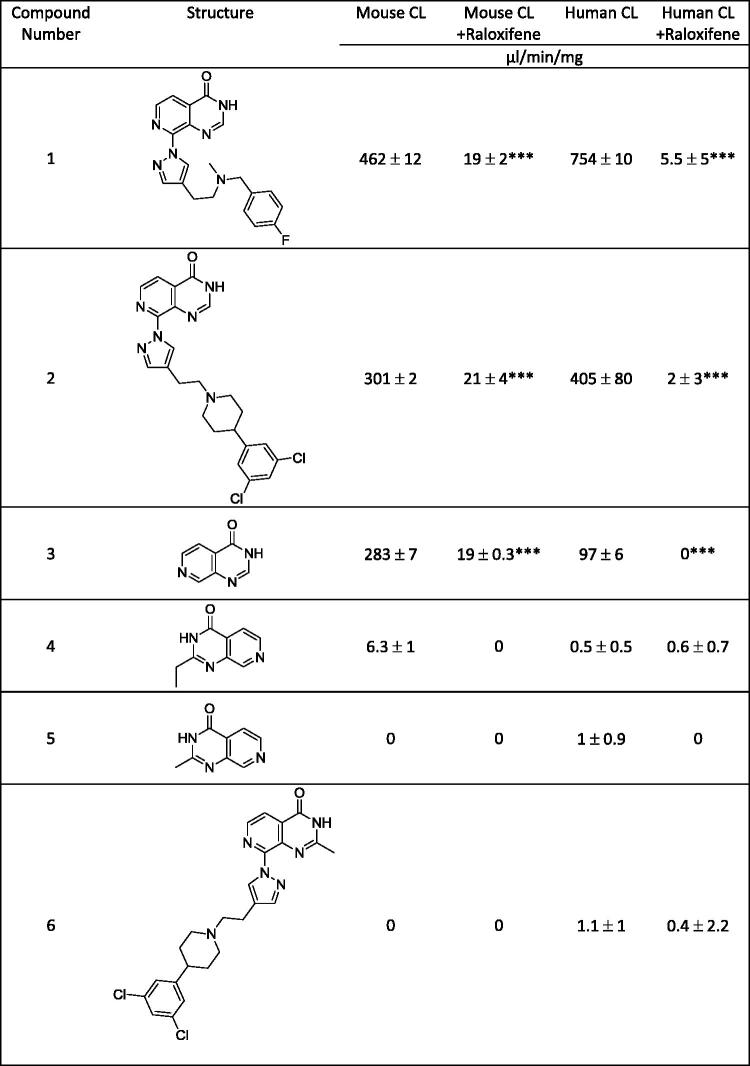
Structure of a number of pyrido[3,4-*d*]pyrimidin-4(3*H*)-one derivatives and clearance in mouse and human cytosol following incubation of 1μM compound in the absence and presence of the aldehyde oxidase inhibitor raloxifene. Values are means ± SD of *n* = 3 replicate analysis. Statistical analysis carried out with paired *t* test comparing clearance with and without inhibitor. ***p < 0.001.

In order to characterise the metabolite(s) of compound **1**, we evaluated the mass spectrometric profile of the cytosolic incubation in comparison with the control cytosol by full scan mass spectrometric analysis. We identified a strong signal at M + 16 (m/z 395.17) consistent with an oxidation product. Fragmentation of the parent (m/z 379.17) and of this metabolite (m/z 395.17) in the mass spectrometer revealed oxidation on the pyrido[3,4-*d*]pyrimidin-4(3*H*)-one core scaffold (m/z 162.03 corresponding to m/z 146.04 + 16). Further fragmentation of the pyrido[3,4-*d*]pyrimidin-4(3*H*)-one was not possible and the exact position of the hydroxylation on the core could not be established by this method (Figure 2). We therefore separated and isolated the metabolite by liquid chromatography to carry out ^1^H NMR analysis of both the parent compound **1** and its metabolites. The ^1^H NMR traces (Figure 3 and supplemental Figure) show that protons C5 and C6 are still present in the metabolite suggesting that that oxidation has occurred at C2 of the pyrido[3,4-*d*]pyrimidin-4(3*H*)-one scaffold. We evaluated both the mouse and human AO clearance of the parent pyrido[3,4-*d*]pyrimidin-4(3*H*)-one scaffold (compound **3**) that lacks any substituent, and its C2-substituted derivatives (compounds **4** and **5**, Figure 4). In addition, we tested an additional pair of C8-substituted compounds, **2** and its C2-methyl counterpart **6**. Consistent with our previous observations regarding compound **1**, the cytosolic clearance of C2-unsubstituted analogues **2** and **3** was high (>90 ml/min/mg protein), but it was significantly reduced by the addition of raloxifene (Figure 4). In contrast, all C2-substituted derivatives (compounds **4**, **5** and **6**) were stable in cytosolic incubations (Figure 4).

**Figure 5.**
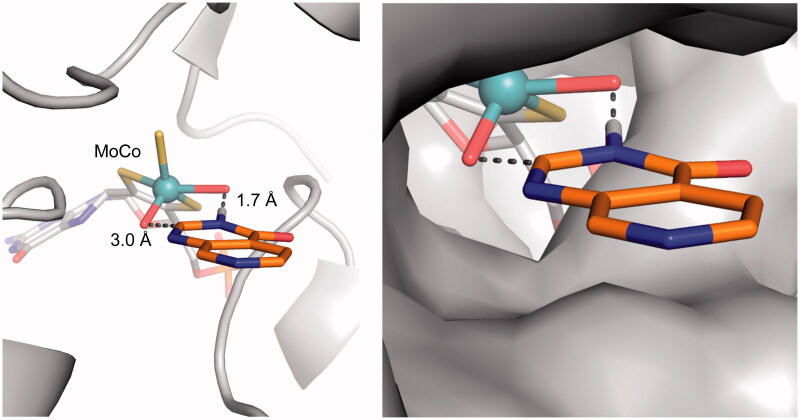
Proposed binding mode of compound **3** (orange sticks) in the human hAOX1 substrate binding site (PDB code 4uhw, grey). Cartoon representation of the protein with the proposed binding mode of **3** to the metal-coordinating oxygen atoms within the molybdenum cofactor MoCo (*left*); Surface representation illustrating the proposed binding mode (*right*).

These results show that the pyrido[3,4-*d*]pyrimidin-4(3*H*)-one scaffold is a substrate for aldehyde oxidase at the C2-position, and that the introduction of a C2-substituent blocks this AO-mediated metabolism. Since the crystal structure of human aldehyde oxidase 1 (hAOX1) was recently published (Coelho et al., 2015), we attempted to predict the binding modes of our pyrido[3,4-*d*]pyrimidin-4(3*H*)-one–based JmjC histone lysine demethylase inhibitors in the hAOX1 substrate binding site. Using the crystal structure of substrate-free hAOX1 (PDB code 4uhw), molecular docking studies of the core scaffold **3** suggested a binding mode that involved cofactor-ligand interactions mediated *via* the 3-position NH of the pyrido[3,4-*d*]pyrimidin-4(3*H*)-one scaffold with the 3 position hydrogen atom at 1.7 Å from one of the metal-coordinating oxygen atoms within the molybdenum cofactor (MoCo). In this binding mode, the carbon atom at the C2-position of the pyrido[3,4-*d*]pyrimidin-4(3*H*)-one scaffold was 3.0 Å away from the other metal-coordinating oxygen atom of MoCo and would be reasonably positioned for nucleophilic oxidation (Alfaro & Jones, 2008). On the contrary, this proposed binding mode of scaffold **3** would not be compatible for exemplars of C2-substituted analogues including compounds **4** and **5** due to steric clash of the alkyl substituent with the AO protein (Figure 5). This observation provided a structural hypothesis explaining why C2-substituted pyrido[3,4-d]pyrimidin-4(3H)-ones are not susceptible to AO-mediated metabolism.

**Figure 6.**
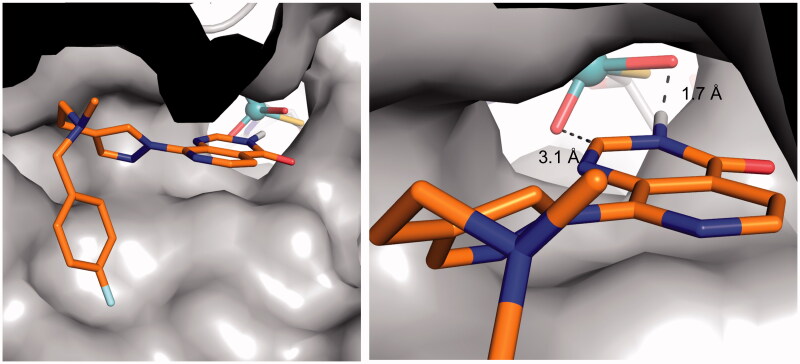
Proposed binding mode of compound **1** (orange sticks) in the human hAOX1 substrate binding site (PDB code 4uhw, grey surface), showing that the bulky C8-substituent can be accommodated in the AO protein. (*left*); close-up illustrating the proposed binding mode interactions (*right*).

To further evaluate the relevance of this proposed binding mode for the pyrido[3,4-*d*]pyrimidin-4(3*H*)-one-based demethylase inhibitor series, compound **1** was modelled in the hAOX1 binding site. The proposed binding mode of compound **1** *via* the pyrido[3,4-*d*]pyrimidin-4(3*H*)-one scaffold was found to be compatible with the accommodation of its bulky C8 substituent in hAOX1 binding site. (Figure 6). These modelling results suggest a binding mode of the pyrido[3,4-*d*]pyrimidin-4(3*H*)-one scaffold that is consistent with a hAOX1 oxidation mechanism at the C2-position and provide a structural insight to explain our observation that introduction of a C2-substituent on the pyrido[3,4-*d*]pyrimidin-4(3*H*)-one scaffold can abrogate AO-mediated metabolism in this chemical series.

## Discussion

Aldehyde oxidase (AO) is a molybdenum-containing enzyme located in the cytosolic compartment of many cells and tissue types and is present in all vertebrates (Carpani et al., 1990; Falciani et al., 1994; Garattini et al., 2008). The fact that compound **1** is a substrate of mouse AO explains why mouse microsomal clearance failed to predict the *in vivo* clearance of this compound. The metabolism of AO substrates has been shown to vary greatly among species with high expression in monkey, low expression in rat and minimal in dogs (Dalgaard, 2015). Various AO isoforms have been described: in humans, only one *AOX1* functional gene is present, although several *AOX1* orthologs are found in most mammals with four in rodents (Terao et al., 2016; Coelho et al., 2012). Mammalian *AOX* gene products are expressed in a tissue-specific manner in different organisms. For example in humans, AOX1 is expressed predominantly in the liver, but detectable amounts are also found in lung and kidney. All these factors impact negatively on the potential accuracy of predictions of human pharmacokinetics of AO substrates (Pryde et al., 2010; Sanoh et al., 2015). Consequently, we decided to understand the basis of the metabolism of compound **1** by human aldehyde oxidase 1 to ascertain if it could be blocked. Our studies established that AO-mediated oxidation occurs at the C2-position of the pyrido[3,4-*d*]pyrimidin-4(3*H*)-one scaffold, which is consistent with literature reports showing AO-mediated nucleophilic attack of an oxygen atom on the electron deficient carbon atoms adjacent to nitrogen of certain aromatic azaheterocycles (Dalvie & Zientek, 2015). The introduction of a C2-alkyl substituent (compounds **4**, **5** and **6**) was sufficient to block AO-mediated metabolism. Subsequent molecular modelling studies predicted ligand-binding modes of core scaffold **3,** and other elaborated analogues including compound **1,** that were compatible with the observed oxidation at the C2 position of the pyrido[3,4-*d*]pyrimidin-4(3*H*)-one scaffold. On the contrary, steric clashes with the protein were predicted for C2-substituted pyrido[3,4-*d*]pyrimidin-4(3*H*)-one derivatives, giving a structural explanation for the lack of AO-mediated metabolism on these compounds.

Compounds based on the pyrido[2,3-*d*]pyrimidin-7-one scaffold such as the p38 kinase inhibitor 6-(2,4-difluorophenoxy)-2-[(1-hydroxypropan-2-yl)amino]-8-(2-hydroxypropyl)-7H,8H-pyrido[2,3-d]pyrimidin-7-one have been shown to be extensively metabolised by human aldehyde oxidase into a 4-hydroxylated metabolite (Zhang et al., 2011). In this instance, the compound which was predicted to have a 6 h half-life in man failed in Phase 1 clinical trial with a half-life of 0.7 h. It is therefore highly desirable to obtain compounds that are not substrates for the AO-mediated metabolism.

In conclusion, our data show that the C2 position in pyrido[3,4-*d*]pyrimidin-4(3*H*)-one-based derivatives is vulnerable to metabolism mediated by AO in both mice and human. Blocking this susceptibility to AO-mediated metabolism at the C2 position should therefore be considered in developing pyrido[3,4-*d*]pyrimidin-4(3*H*)-one-based tool compounds for *in vivo* studies, and also in exploring the potential of such compounds as anticancer agents.

## Supplementary Material

IXEN_1230245_Supplementary_Material.pdf

## Notes

**Supplementary material available online.**
