# Supplementary material for: Pyrido[3,4-d]pyrimidin-4(3H)-one metabolism mediated by aldehyde oxidase is blocked by C2-substitution
Source: Xenobiotica. 2016 Oct 26;47(9):771–7. doi: 10.1080/00498254.2016.1230245 (PMC5526139; doi:10.1080/00498254.2016.1230245)

Chemistry: Commercially available starting materials, reagents and anhydrous solvents were used as supplied. Flash column chromatography was performed using Merck silica gel 60 (0.025 – 0.04 mm). Thin layer chromatography was performed using Merck Millipore TLC silica gel 60 F_{254} aluminium sheets and visualised by UV (254 & 280 nm), iodine and $KMnO_4$. Column chromatography was also performed on a FlashMaster personal unit using isolute Flash silica columns or a Biotage Isolera purification system using Biotage KP-SNAP cartridges. Ion exchange chromatography was performed using acidic Isolute Flash SCX-II cartridges or basic Isolute Flash NH_2 cartridges. ^{1}H NMR spectra were recorded on either a Bruker Avance-500 or Bruker Avance-400 NMR machine. Samples were prepared as solutions in a deuterated solvent and referenced to the appropriate internal non-deuterated solvent peak or tetramethylsilane. Chemical shifts were recorded in ppm (δ) downfield of tetramethylsilane.

LC/MS and HRMS analysis: Analysis was performed on a Waters Acquity UPLC and diode array detector coupled to a Waters G2 QToF mass spectrometer fitted with a multimode ESI/APCI source. Method A: Analytical separation was carried out at 30 °C on a Phenomenex Kinetex C18 column (30 x 2.1 mm, 2.6u, 100A) using a flow rate of 0.5 mL/min in a 2 minute gradient elution with detection at 254 nm. The mobile phase was a mixture of methanol (solvent A) and water (solvent B), both containing formic acid at 0.1%. Gradient elution was as follows: 10:90 (A/B) to 90:10 (A/B) over 1.25 min, 90:10 (A/B) for 0.5 min, and then reversion back to 10:90 (A/B) over 0.15 min, finally 10:90 (A/B) for 0.1 min. Method B: Analytical separation was carried out at 30°C on a Phenomenex Kinetex C18 column (30 x 2.1 mm, 2.6u, 100A) using a flow rate of 0.3 mL/min in a 4 minute gradient elution with detection at 254 nm. The mobile phase was a mixture of methanol (solvent A) and water (solvent

B), both containing formic acid at 0.1%. Gradient elution was as follows: 10:90 (A/B) to 90:10 (A/B) over 3 min, 90:10 (A/B) for 0.5 min, and then reversion back to 10:90 (A/B) over 0.3 min, finally 10:90 (A/B) for 0.2 min.

LC/MS and HRMS analysis was also performed on an Agilent 1200 series HPLC and diode array detector coupled to a 6210 time of flight mass spectrometer with dual multimode APCI/ESI source. Method C: Analytical separation was carried out at 30°C on a Merck Chromolith Flash column (RP-18e, 25 x 2 mm) using a flow rate of 0.75 mL/min in a 4 minute gradient elution with detection at 254 nm. The mobile phase was a mixture of methanol (solvent A) and water (solvent B), both containing formic acid at 0.1%. Gradient elution was as follows: 5:95 (A/B) to 100:0 (A/B) over 1 min, 100:0 (A/B) for 2.5 min, and then reversion back to 5:95 (A/B) over 0.1 min, finally 5:95 (A/B) for 0.4 min.

LC-HRMS method B referenced to Leucine Enkephalin fragment ion $[M+H]^+$ 397.1876, and LC-HRMS method C referenced to caffeine $[M+H]^+$ 195.087652; reserpine $[M+H]^+$ 609.280657 or hexakis (2,2-difluroethoxy)phosphazene $[M+H]^+$ 622.02896.

2-Ethylpyrido[3,4-*d*]pyrimidin-4(3*H*)-one (compound 4)

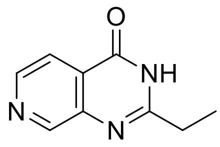

A solution of 3-aminoisonicotinamide (0.10 g, 0.73 mmol) in 1,1,1-triethoxypropane (5 mL) was stirred at 160 °C in a sealed microwave tube for 3 h. It was then cooled to room temperature and the brown solid that formed was filtered, washed with ether

(2 x 2 mL) and dried ($MgSO_4$). The pure product was isolated as a pale brown powder (0.052 g, 41%). ^{1}H NMR (500 MHz, DMSO-d_6) δ 1.25 (t, J = 7.5 Hz, 3H), 2.65 (q, J = 7.5 Hz, 2H), 7.89 (d, J = 5.2 Hz, 1H), 8.59 (d, J = 5.2 Hz, 1H), 8.98 (s, 1H), 12.51 (br s, 1H); LC-MS (Method A, ESI, *m/z*) t_R = 0.71 min – 176 $(M+H)^+$; HRMS (Method B): found 176.0827; calculated for $C_9H_{10}N_3O$ $(M+H)^+$ 176.0824.

2-Methylpyrido[3,4-*d*]pyrimidin-4(3*H*)-one (Compound 5)

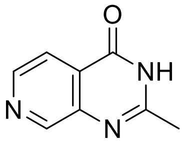

A solution of 3-aminoisonicotinamide (0.10 g, 0.73 mmol) in 1,1,1-triethoxyethane (5 mL) was stirred at 160 ^{o}C in a sealed microwave tube for 3 h. It was then cooled to room temperature and the brown solid that formed was filtered, washed with ether (2 x 2 mL) and dried ($MgSO_4$). The pure product was isolated as a pale brown powder (0.055 g, 47%). ^{1}H NMR (500 MHz, DMSO-d_6) δ 2.38 (s, 3H), 7.88 (d, J = 5.2 Hz, 1H), 8.58 (d, J = 5.2 Hz, 1H), 8.94 (s, 1H), 12.54 (br s, 1H); HRMS (Method B) t_R = 0.98 min; found 162.0674; calculated for $C_8H_8N_3O$ $(M+H)^+$ 162.0667.

8-Chloro-2-methylpyrido[3,4-*d*]pyrimidin-4(3*H*)-one (intermediated for the synthesis of compound **6**)

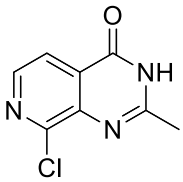

A solution of 3-amino-2-chloroisonicotinamide (0.20 g, 1.17 mmol) in 1,1,1-triethoxyethane (5 mL) was stirred at 160 ^{o}C in a sealed microwave tube for 2 h. It was cooled to room temperature and the solid that formed was filtered, washed with

ether (2 x 2 mL) and dried. The pure product was isolated as a yellow powder (0.098 g, 43%). ^{1}H NMR (500 MHz, DMSO-d_6) 2.42 (s, 3H), 7.90 (d, J = 5.1 Hz, 1H), 8.35 (d, J = 5.1 Hz, 1H), 12.78 (s, 1H); LC-MS (Method A, ESI, *m/z*) t_R = 0.74 min – 196 $(M+H)^+$.

8-Chloro-2-methyl-3-((2-(trimethylsilyl)ethoxy)methyl)pyrido[3,4-*d*]pyrimidin-4(3*H*)-one (intermediate for the synthesis of compound **6**)

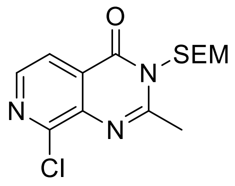

8-Chloro-2-methylpyrido[3,4-*d*]pyrimidin-4(3*H*)-one (0.098 g, 0.50 mmol, 1 equiv.) was dissolved in dry DMF (1 mL, 0.5 M). The solution was heated to 60 ˚C and potassium carbonate (0.138 g, 1.0 mmol, 2 equiv.) was added. After 5 minutes of stirring, (2-(chloromethoxy)ethyl)trimethylsilane (0.10 g, 0.6 mmol, 1.2 equiv.) was slowly added. Stirring was continued at 60 ˚C for 3 h, the reaction mixture was cooled to room temperature and EtOAc (20 mL) was added. The heterogeneous mixture was washed with water (2 × 20 mL), dried (Na_2SO_4) and concentrated *in vacuo*. The crude product was purified by silica gel column chromatography (3% EtOAc in CH_2Cl_2) to provide the title compound as a white powder (0.10 g, 61%); ^{1}H NMR (500 MHz, $CDCl_3$) δ 0.01 (s, 9H), 0.93 – 0.98 (m, 2H), 2.80 (s, 3H), 3.66 – 3.69 (m, 2H), 5.57 (s, 2H), 7.96 (d, J = 5.1 Hz, 1H), 8.38 (d, J = 5.1 Hz, 1 H), LC - MS (Method A; ESI, *m/z*) t_R = 1.51 min – 268, 270 [$(M- SEM +TMS)^+$, Cl isotopic pattern)].

8-(4-(2-((*tert*-Butyldimethylsilyl)oxy)ethyl)-1*H*-pyrazol-1-yl)-2-methyl-3-((2-(trimethylsilyl)ethoxy)methyl)pyrido[3,4-*d*]pyrimidin-4(3*H*)-one (intermediate for the synthesis of compound **6**)

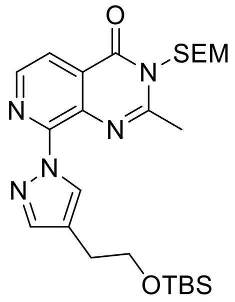

Cesium carbonate (0.20 g, 0.61 mmol, 2 equiv.) and 4-(2-((*tert*-butyldimethylsilyl)oxy)ethyl)-1*H*-pyrazole (Bavetsias, V. *et al*, *J. Med. Chem.* 2016, *59*, 1388-1409) (0.083 g, 0.37 mmol, 1.2 equiv.) were added to a microwave vial containing 8-chloro-2-methyl-3-((2-(trimethylsilyl)-ethoxy)methyl)pyrido[3,4-*d*]pyrimidin-4(3*H*)-one (0.10 g, 0.31 mmol, 1 equiv) equipped with a stirrer bar. This was sealed, evacuated and flushed with N_2. Anhydrous MeCN (2 mL) was added to the vial which was sealed and the reaction mixture stirred at 110 ^{o}C for 18 h. Solids were removed by filtration and rinsed with CH_2Cl_2 (3 × 5 mL). The filtrate was concentrated *in vacuo* and purified by silica column chromatography (30% EtOAc in cyclohexane) to give the title product as a pale yellow solid (0.063 g, 40%); ^{1}H NMR (500 MHz, $CDCl_3$) δ 0.03 (s, 9H), 0.06 (s, 6H), 0.90 (s, 9H), 0.92-0.98 (m, 2H), 2.78 (s, 3H), 2.81 (t, *J* = 6.7 Hz, 2H), 3.69-3.72 (m, 2H), 3.84 (t, *J* = 6.7 Hz, 2H), 5.60 (s, 2H), 7.77 (s, 1H), 7.98 (d, *J* = 5.1 Hz, 1H), 8.58 (s, 1H), 8.75 (s, 1H) ; LC - MS (Method C; ESI, *m/z*) t_R = 3.64 min – 516 $(M+H)^+$.

8-(4-(2-Hydroxyethyl)-1*H*-pyrazol-1-yl)-2-methyl-3-((2-(trimethylsilyl)ethoxy)methyl)pyrido[3,4-*d*]pyrimidin-4(3*H*)-one (intermediate for the synthesis of compound **6**)

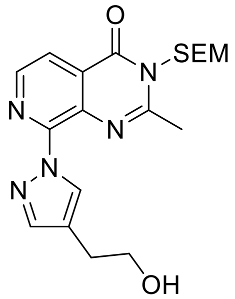

Hydrochloric acid (1 M, 1.2 mL, 10 equiv.) was added to a solution of 8-(4-(2-((tert-butyldimethylsilyl)oxy)ethyl)-1*H*-pyrazol-1-yl)-2-methyl-3-((2-(trimethylsilyl)ethoxy)methyl)pyrido[3,4-*d*]pyrimidin-4(3*H*)-one (0.063 g, 0.12 mmol, 1 equiv.) in MeOH (1 mL, 0.1 M) at 0 °C. The reaction was stirred at 0 °C for 5 min, then diluted with EtOAc (10 mL), washed with saturated $NaHCO_3$ solution (5 mL), saturated brine solution (5 mL), dried over $MgSO_4$ and concentrated *in vacuo* to give the product as a white solid (0.033g, 69%); ^{1}H NMR (500 MHz, $CDCl_3$) δ 0.03 (s, 9H), 0.94 - 0.98 (m, 2H), 2.76 (s, 3H), 2.86 (t, *J* = 6.3 Hz, 2H), 3.68-3.72 (m, 2H), 3.88 (t, *J* = 6.3 Hz, 2H), 5.59 (s, 2H), 7.76 (s, 1H), 7.97 (d, *J* = 5.1 Hz, 1H), 8.56 (d, *J* = 5.1 Hz, 1H), 8.75 (s, 1H), OH signal not observed; LC - MS (Method C; ESI, *m/z*) t_R = 3.04 min – 402 $(M+H)^+$.

8-(4-(2-(4-(3,5-Dichlorophenyl)piperidin-1-yl)ethyl)-1*H*-pyrazol-1-yl)-2-methyl-3-((2-(trimethylsilyl)ethoxy)methyl)pyrido[3,4-*d*]pyrimidin-4(3*H*)-one (intermediate for the synthesis of compound **6**)

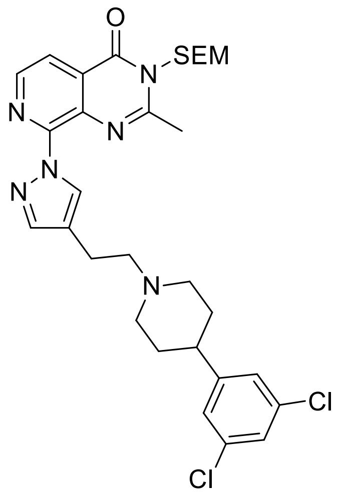

Methanesulfonic anhydride (0.022 g, 0.12 mmol, 1.5 equiv.) was added in one portion to a solution of 8-(4-(2-hydroxyethyl)-1*H*-pyrazol-1-yl)-2-methyl-3-((2-(trimethylsilyl)ethoxy)methyl)pyrido[3,4-*d*]pyrimidin-4(3*H*)-one

(0.033 g, 0.08 mmol, 1 equiv.), and triethylamine (0.016 g, 0.16 mmol, 2 equiv.) in anhydrous CH_2Cl_2 (1 mL) at 0 °C under N_2. The reaction mixture was stirred for 15 min at 0 °C and monitored by LCMS. When the reaction had gone to completion, the reaction mixture was quenched with a saturated solution $NaHCO_3$ (10 mL) and extracted with CH_2Cl_2 (3 × 15 mL). The combined organic layers were washed with saturated brine solution (30 mL), dried over $MgSO_4$ and concentrated *in vacuo* to give 2-(1-(2-methyl-4-oxo-3-((2-(trimethylsilyl)ethoxy)methyl)-3,4-dihydropyrido[3,4-*d*]pyrimidin-8-yl)-1*H*-pyrazol-4-yl)ethyl methanesulfonate as a pale yellow oil (0.035 g, 87%). This material was used in the next step without further purification. A solution of the mesylate (0.035 g, 0.073 mmol), 4-(3,5-dichlorophenyl)piperidine (0.016 g, 0.073 mmol) and triethylamine (0.014 g, 0.146 mmol) in dry DMF (1 mL) was heated in a sealed microwave tube at 60 ^{o}C for 3 days. Volatiles were removed *in vacuo* and the residue purified on a silica column (5% of a solution of 7N ammonia in methanol in ethyl acetate) to afford the title compound as a colorless oil (0.013 g,

29%). ^{1}H NMR (500 MHz, $CDCl_3$) δ 0.00 (s, 9H), 0.95-0.99 (m, 2H), 1.25 – 1.29 (m, 2H), 1.89 – 1.97 (m, 2H), 2.18 – 2.24 (m, 2H), 2.40 – 2.63 (m, 2H), 2.80 (s, 3H), 2.82 – 2.90 (m, 1H), 2.79 – 2.91 (m, 2H), 3.24 (br d, *J* = 11.4 Hz, 2H), 3.70 – 3.73 (m, 2H), 5.61 (s, 2H), 7.13 (d, *J* = 1.9 Hz, 2H), 7.21 (t, *J* = 1.9 Hz, 1H), 7.76 (s, 1H), 7.99 (d, *J* = 5.1 Hz, 1H), 8.58 (d, *J* = 5.1 Hz, 1H), 8.76 (s, 1H); LC - MS (Method C; ESI, *m/z*) t_R = 2.95 min – 613, 615, 617 [$(M+H)^+$, Cl_2 isotopic pattern].

8-(4-(2-(4-(3,5-Dichlorophenyl)piperidin-1-yl)ethyl)-1*H*-pyrazol-1-yl)-2-methylpyrido[3,4-*d*]pyrimidin-4(3*H*)-one (Compound 6)

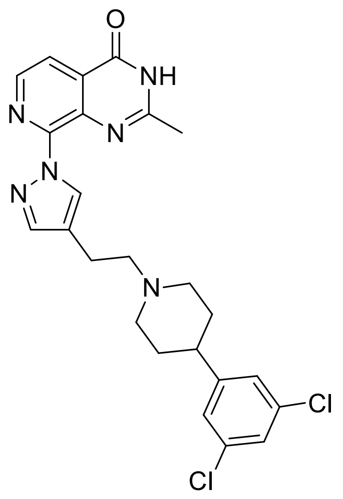

Hydrochloric acid (6 M, 1 mL) was added to a solution of 8-(4-(2-(4-(3,5-dichlorophenyl)piperidin-1-yl)ethyl)-1*H*-pyrazol-1-yl)-2-methyl-3-((2-(trimethylsilyl)ethoxy)methyl)pyrido[3,4-*d*]pyrimidin-4(3*H*)-one (0.013 g, 0.021 mmol) in THF (1 mL) and the reaction mixture was heated at 60 ^oC for 2 h in a sealed microwave tube. Volatiles were removed *in vacuo* and the residue purified on KP-NH snap cartridge (40% EtOH in CH_2Cl_2) to give the product as a white solid (0.0028 g, 27%) ^{1}H NMR (500 MHz, MeOH-d_4) δ 1.80 (qd, *J* = 12.4, 3.5 Hz, 2H), 1.88 (d, *J* = 12.4 Hz, 2H), 2.25 (dt, *J* = 11.6, 1.6 Hz, 2H), 2.52 (s, 3H), 2.60-2.67 (m, 1H), 2.74 – 2.76 (m, 2H), 2.87 – 2.90 (m, 2H), 3.23 (br d, *J* = 11.3 Hz, 2H), 7.25 (d, *J* = 1.9 Hz,

2H), 7.28 (t, *J* = 1.9 Hz, 1H), 7.80 (s, 1H), 8.01 (d, *J* = 5.1 Hz, 1H), 8.49 (d, *J* = 5.1 Hz, 1H), 8.80 (s, 1H) ; LC- MS (Method A; ESI, *m/z*) t_R = 1.03 min – 483, 485, 487 [$(M+H)^+$, Cl_2 isotopic pattern]; HRMS (Method B): found 483.1447; calculated for $C_{24}H_{25}Cl_2N_6O$ $(M+H)^+$ 483.1461.

Supplemental Figure

Compound1 ^{1}H NMR (500 MHz, DMSO-d6) δ 8.48 (s, 1H, 16), 8.44 (d, J = 5.1 Hz, 1H, 1), 8.29 (s, 1H, 8), 7.92 (d, J = 5.1 Hz, 1H, 6), 7.66 (s, 1H, 14), 7.34 (dd, J = 8.4, 5.7 Hz, 2H, 23, 27), 7.12 (t, J = 8.8 Hz, 2H, 24, 26), 3.53 (s, 2H, 21), 2.73 (t, J = 7.3 Hz, 2H, 17), 2.61 (t, J = 7.4 Hz, 2H, 18), 2.18 (s, 3H, 20).

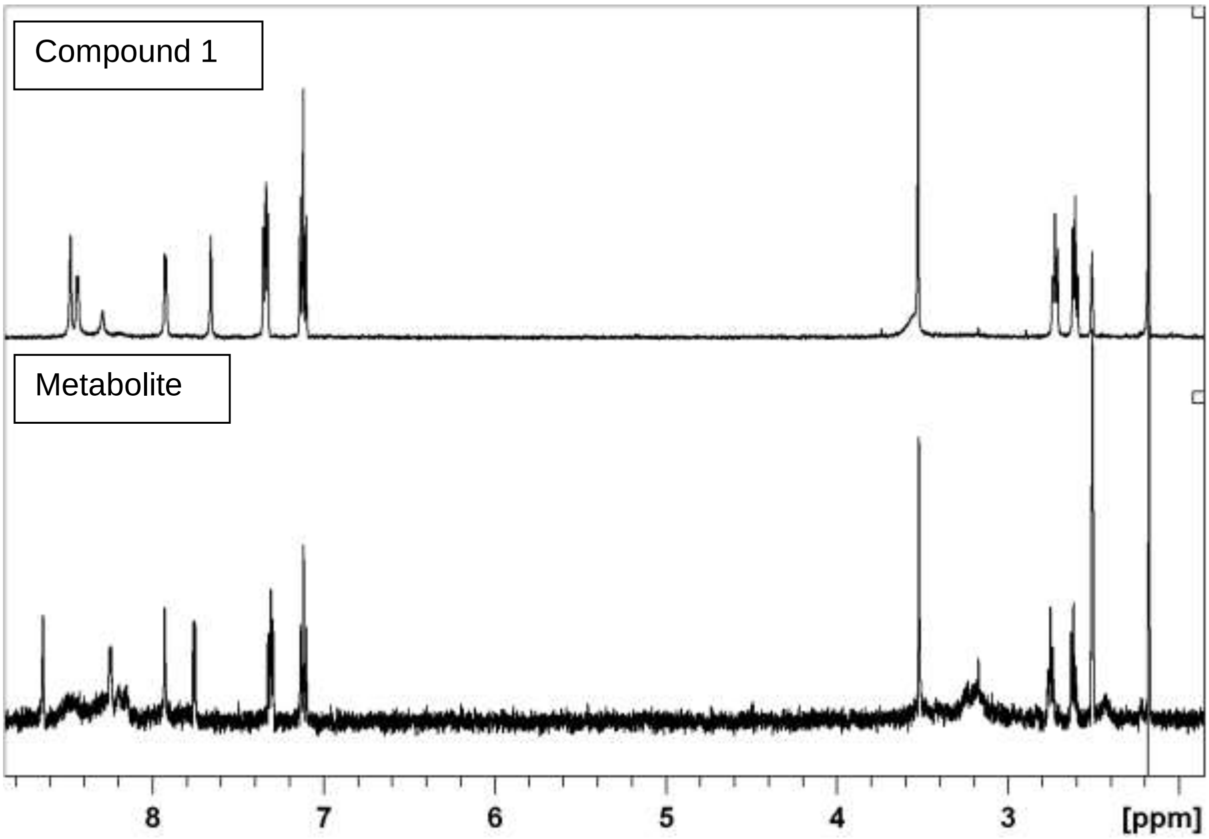

Supplement: IXEN_1230245_Supplementary_Material.pdf [file IXEN_A_1230245_SM2023.pdf]
